# Tissue Microarray: A rapidly evolving diagnostic and research tool

**DOI:** 10.4103/0256-4947.51806

**Published:** 2009

**Authors:** Nazar M.T. Jawhar

**Affiliations:** Department of Pathology, Ninevah Medical College, University of Mosul, Iraq

## Abstract

Tissue microarray is a recent innovation in the field of pathology. A microarray contains many small representative tissue samples from hundreds of different cases assembled on a single histologic slide, and therefore allows high throughput analysis of multiple specimens at the same time. Tissue microarrays are paraffin blocks produced by extracting cylindrical tissue cores from different paraffin donor blocks and re-embedding these into a single recipient (microarray) block at defined array coordinates. Using this technique, up to 1000 or more tissue samples can be arrayed into a single paraffin block. It can permit simultaneous analysis of molecular targets at the DNA, mRNA, and protein levels under identical, standardized conditions on a single glass slide, and also provide maximal preservation and use of limited and irreplaceable archival tissue samples. This versatile technique, in which data analysis is automated facilitates retrospective and prospective human tissue studies. It is a practical and effective tool for high-throughput molecular analysis of tissues that is helping to identify new diagnostic and prognostic markers and targets in human cancers, and has a range of potential applications in basic research, prognostic oncology and drug discovery. This article summarizes the technical aspects of tissue microarray construction and sectioning, advantages, application, and limitations.

Recent advances in the field of human molecular genetics have revealed gene-based disease mechanisms in many areas of medicine. The study of new prognostic and diagnostic markers in large numbers of clinical specimens is an important step in translating the new findings from basic science to clinill cal practice.^1^ The investigation of the pathogenesis and progression of diseases such as cancer has been revolutionized with the increased use of new molecular biology techniques.^2^ Elucidating the fundamental molecular mechanisms that are involved in the stepwise progression from normal tissues to malignant tumors is essential in our knowledge of cancers, and should ultimately lead to improved methods of detection, treatment, and cures for cancers.^3^ Studies on clinical tissue have identified multiple novel markers, primarily at the gene level.^4^ The validation of these markers using the standard histopathological techniques is time consuming, and labor intensive and costly, particularly when multiple markers are tested on numerous specimens.^4^

Tissue microarray is a recent innovation in the field of pathology that is expected to overcome these significant problems. The method was designed as a high-throughput molecular biology technique for researchers that allows for assessment of expression of interesting candidate diseaselrelated genes or gene products simultaneously on hundreds of tissue samples.^5^ It also allows parallel molecular profiling of clinical samples at the DNA, RNA, and protein level. This technique enables pathologists to perform large-scale analyses using immunohistochemistry, fluorescence in situ hybridization (FISH), or RNA in situ hybridization (ISH) at substantially faster and at markedly lower costs compared with conventional approaches.^1^,^6^ This technology should not be confused with DNA microarrays where each tiny spot represents a unique cloned cDNA or oligonucleotide. In tissue microarrays, the spots are larger and contain small histologic sections from unique tissues or tumors.

This article provides a short review of this increasingly popular technology, focusing on several technical aspects of tissue microarray construction.This technology is relatively new and its use in developing countries has never been limited.^7^–^9^ The technique was first reported 20 years ago by Battifora who described a “sausage block” method in which he wrapped 1 mm thick ‘rods’ of different tissues in a sheet of small intestine which was then embedded in a paraffin block and from which numerous sections were cut and examined.^10^ The array format was first conceived by Wan and colleagues in 1987.^11^ Although this technique conferred the significant advantage of simultaneously examining multiple tissue specimens under identical conditions, the inability to satisfactorily identify individual ‘rods’ limited any meaningful interpretation. These limitations were addressed subsequently, and in 1998, Kononen et al invented a device for rapid and accurate construction of tissue microarrays in a manner that is easily accessible to most pathology labs. The invention of this device and its commercialization led to a dramatic increase in the popularity and utility of the technique.^12^

## Tissue microarray technique

**Figure 1:**
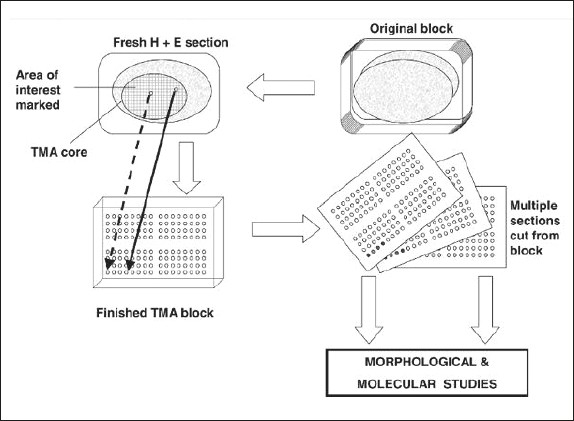
Construction of a tissue microarray (TMA).

Microarray is a technique for organizing minute amounts of biological samples on a solid support.^13^ Tissue microarrays are composite paraffin blocks constructed by extracting cylindrical tissue core “biopsies” from different paraffin donor blocks and re-embedding these into a single recipient (microarray) block at defined array coordinates.^14^,^15^ At first, the donor blocks (invariably stored paraffin blocks) are retrieved and sectioned to produce standard microscopic slides that are stained with hematoxylin and eosin. An experienced pathologist examines the slides to mark the area of interest, which is commonly an area of cancer (Figure 1), after which the samples can be arrayed.^4^

**Figure 2:**
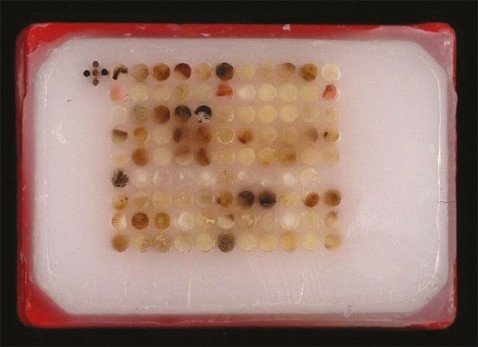
Tissue microarray block.

**Figure 3:**
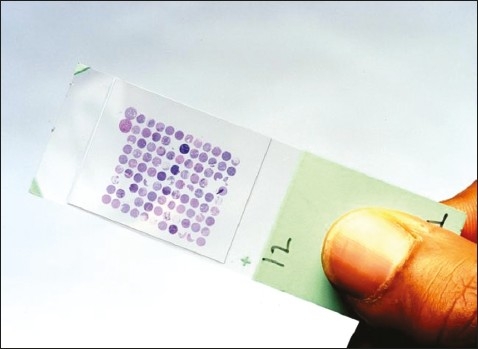
A tissue microarray slide.

A tissue microarray instrument (for example, Beecher Instruments, Winsconsin, USA, *www.beecher instruments.com*) is used to acquire a tissue core from the donor block.^16^ This core is then placed in an empty paraffin block—the recipient block (Figure 2).^16^ The current Beecher Instruments arraying device is designed to produce sample circular spots that are 0.6 mm in diameter at a spacing of 0.7-0.8 mm.^17^ The surface area of each sample is 0.282 mm^2^, or in pathologists' terms, about the size of 2-3 high power fields. The number of spots on a single slide is variable depending on the array design; the current comfortable maximum with the 0.6 mm needle is about 600 spots per standard glass microscope slide.^15^ The core is placed at a specifically assigned coordinate (X-Y guide), which is accurately recorded, typically on a spreadsheet, such as Microsoft Excel.^4^ The sampling process can then be repeated many times from different donor blocks until hundreds, or even thousands, of cores are placed into one recipient block, producing the final tissue microarray block (Figure 2). Using a microtome, 5 μm sections are cut from the tisll sue microarray blocks to generate tissue microarray slides for molecular and immunohistochemical analyses (Figure 3).^16^

New technologies are under development that may allow as many as 2000 or more sections per slide. Using this method, an entire cohort of cases can be analyzed by staining just one or two master array slides, instead of staining hundreds of conventional slides.^18^ Yet each spot on the array is similar to a conventional slide in that complete demographic and outcome information is maintained for each case so that rigorous statistical analysis can be done as rapidly as the arrays are analyzed.^15^

### Advantages and applications of tissue microarrays

There are numerous advantages of tissue microarray over standard techniques, including:

*Amplification of a scarce resource*. A standard histologic section is about 3-5 mm thick, with variation depending on the submitting pathologist or technician. After use for primary diagnosis, the sections can be cut 50-100 times depending on the care and skill of the sectioning technician. Thus, on average, each archived block might yield material for a maximum of 100 assays.^13^ If this same block is processed for optimal microarray construction it could routinely be needle biopsied 200-300 times or more depending on the size of the tumor in the original block. Once tissue microarrays are constructed, they can be judiciously sectioned to maximize the number of sections cut from an array. The sectioning process uses a tape-based sectioning aid (from Instrumedics Inc., Missouri, USA, *www.instrumedics.com*) that allows cutting of thinner sections. Optimal sectioning of arrays is obtained with about 2-3 μm sections. Thus, instead of 50-100 conventional sections or samples for analysis from one tissue biopsy, the microarray technique could produce material for 500 000 assays (assuming 250 biopsies per section times 2000 2.5-μm sections per 5 mm array block) represented as 0.6 mm disks of tissue. Thus this technique essentially amplifies (up to 10 000 fold) the limited tissue resource.^15^

*Simultaneous analysis of very large numbers of specimens.* Tissue microarrays provide high throughput data acquisition. For instance, if a tissue microarray block containing 1000 cores is cut 200 times, this allows for 200 000 individual assays.^19^,^20^

*Experimental uniformity.* With this technology, each tissue sample is treated in an identical manner and microarrays are amenable to a wide range of techniques, including histochemical stains, immunologic stains with either chromogenic or fluorescent visualization, in situ hybridization (including both mRNA ISH and FISH), and even tissue micro-dissection techniques. For each of these protocols, conventional procedures can have substantial slideltolslide variability associated with processing 300 slides (for example, 20 batches of 15 slides). Tissue microarray allows the entire cohort to be analyzed in one batch on a single slide. Thus, variables such as antigen retrieval, temperature, incubation times, washing procedure, and reagent concentration are standardized for the entire cohort.^21^–^23^

*Decreased assay volume, time and cost.* As only a very small amount of reagent (a few μL) is required to analyze an entire cohort, less laboratory personnel are required to perform the experiments. This method has proven to be extremely efficient, of shorter duration, and cost effective, especially with expensive reagents.^4^ This advantage raises the possibility of use of tissue microarrays in screening procedures.

*Does not destroy original block for diagnosis and thus conserves valuable tissue.* There are occasions where the original block must be returned to the patient or donating institution. In these cases the block may be cored a few times without destroying the original tissue block. Then, upon subsequent sectioning, it is still possible to make a diagnosis, even though tissue has been taken for array-based studies.

Tissue microarray has proved to be an effective and efficient tool for assessing quality assurance programs such as intra- and interlaboratory variation in immuno-histochemical and molecular studies. A tissue microarray block can be created from numerous tissue specimens, then sectioned and unstained slides distributed to different laboratories, with each laboratory doing immunostains or molecular tests. Thus, tissue microarray can facilitate the standardization of immunohistochemical, fluorescence in situ hybridization, and other molecular assays, so that results are reproducible between laboratories. The tissue microarray also can be used in internal quality control (eg, quarterly or monthly) to ensure that there is no drift of staining results or reporting within a laboratory. It can also be used for optimization of diagnostic reagents such as monoclonal antibodies and gene probes.^5^ It is believed that the tissue microarray is an improvement over the current practice of using a single strong positive control for quality.^24^–^26^

Tissue microarray facilitates rapid translation of molecular discoveries to clinical applications. The technique has been applied to tumor research (gliomas, breast tumors, lung cancer among others). The development of novel biochip technologies has opened up new possibilities for the high-throughput molecular profiling of human tumors. Novel molecular markers emerging from high-throughput expression surveys can be analyzed on tumor tissue microarray.^27^ Newly identified genes require clinical validation on histopathological specimens for any meaningful outcome. Such validation is best achieved by tissue microarray, as is seen in the analysis of tumor specimens. Depending on this, three categories of tissue microarray can be defined:^4^ (1) With multitumor arrays, many tumor types are sampled, from a diverse set of donor blocks, and arrayed on one recipient tissue microarray block. With this type of tissue microarray, a large group of tumors can then be expeditiously screened for the presence or absence of novel markers.^28^–^30^ (2) For tumor progression arrays, morphological and molecular changes through the different stages of tumor progression, of one particular tumor type, can be assessed in tumor progression tissue microarrays. In prostate cancer, for example, construction of such an array would involve sampling of normal prostate, benign prostatic hyperplasia, prostatic intraepithelial neoplasia and different stages of prostate cancer, from localized disease to metastatic cancer. In one of the most significant papers on prostate cancer recently, a tumor progression array was used to show that the expression of a novel protein, EZH2, correlated with aggressiveness of disease.^31^ (3) In prognostic (patient outcome) arrays, correlation of tissue microarray-derived data with clinical follow-up, to assess prognosis or patient outcome, is of significant interest to clinicians and their patients.^4^ An example is the study of the expression of the EZH2 protein in prostatic cancer. Beside its benefit as a marker of prostate cancer progression, it was shown that the degree of expression of this protein was related to outcome after radical prostatectomy.^31^ Whilst strong expression was associated with recurrence of tumor after surgery in a third of patients, weak EZH2 staining was found in only 9% of individuals with clinical failure. Similar associations have been described in other neoplasms.^32^–^34^

### Tissue heterogeneity and other disadvantages of tissue microarray

One of the most common criticisms of tissue microarray is that the small cores sampled may not be representative of the whole tumor, particularly in heterogenous cancers such as prostate adenocarcinoma and Hodgkin lymphoma.^4^,^13^ However, many groups have shown excellent concordance between tissue microarray spots and whole sections in immunohistochemical studies of multiple tumor types. Comparing the results of whole tissue sections with that of tissue microarray in quality assessment of estrogen receptor status in breast cancer, Parker et al found the results of microarray core for estrogen receptors (ie, positive vs negative) were the same as the results of the whole sections of the tumors in 96% of cases.^24^ Another study that examined validation of tissue microarray technology for immunohistochemical assays, found that the analysis of two core sections from one case was comparable to the analysis of whole tissue sections in more than 95% of cases.^35^ A third study of more than 2000 bladder cancers assessed for histologic grade and proliferative index, information obtained from four 0.6-mm cores per case was highly concordant with that obtained from the whole sections.^36^ These findings demonstrate that intratumoral heterogeneity should not be a major impediment to use of arrays in quality assurance studies. Another minor disadvantage includes the absence of one or more core sections on the immunostained slide.^24^ Although any given histo-spot may be negative or absent on a given array, the statistical power of analysis of hundreds or thousands of cases eliminates the affect of variability of a single data point in the ultimate conclusions.^15^

## Automation

Attempts have been made to automate the process of tissue microarray construction. Various machines are now available (automated tissue arrayer) which may array single and multiple tissue microarray blocks in an even shorter period of time than that performed manually.^37^ Furthermore, digital techniques have taken center stage in clinical medicine as well as in biomedical research. Recently pathology has begun to embrace this digital revolution. The first development was a high throughput microscopic slide scanner that can convert traditional glass microscopic slides into digital images that can be stored, retrieved, shared through the internet and, most importantly, algorithmically analyzed. The other development was the creation of algorithms that can identify and quantitate immunohistochemical staining patterns and specific histological features, such as nuclear size and mitosis. Such an algorithm can be applied to digital images of microarray. Digital pathology and imaging technology that can scan tissue microarrays into “virtual slides” with high resolution and that can analyze their images algorithmically will accelerate the discovery of new predictive biomarkers.^38^

## Conclusion

Tissue microarray is a practical and effective tool for high-throughput molecular analysis of tissues that is helping identify new diagnostic and prognostic markers and targets in human cancers. It has varying degrees of research use and offers a range of potential applications in basic research, prognostic oncology and drug discovery. It is anticipated that tissue microarray will soon become a widely used tool for all types of tissue-based research. Tissue microarray technique will lead to a significant acceleration in the transition of basic research findings into clinical applications.
